# Perceptions of the Feasibility and Practicalities of Text Messaging-Based Infectious Disease Surveillance: A Questionnaire Survey

**DOI:** 10.2196/mhealth.4509

**Published:** 2016-05-25

**Authors:** Linh Thuy Dang, Nguyen Cong Vu, Thiem Dinh Vu, Spencer L James, Peter Katona, Lindsay Katona, Joseph M Rosen, Cuong Kieu Nguyen

**Affiliations:** ^1^ Institute of Population, Health and Development Hanoi Vietnam; ^2^ National Institute of Hygiene and Epidemiology Hanoi Vietnam; ^3^ Geisel School of Medicine Dartmouth College Hanover, NH United States; ^4^ David Geffen School of Medicine University of California, Los Angeles Los Angeles, CA United States; ^5^ College of Osteopathic Medicine University of New England Biddeford, ME United States; ^6^ Thayer School of Engineering Dartmouth College Hanover, NH United States

**Keywords:** SMS, SMS-based, infectious diseases, text messaging, surveillance, Vietnam

## Abstract

**Background:**

In Vietnam, infectious disease surveillance data are collected via a paper-based system through four government tiers leading to a large delay. Meanwhile, mobile phones are abundant and very popular in the country, and known to be a useful tool in health care worldwide. Therefore, there is a great potential for the development of a timely disease surveillance system through the use of mobile phone short message service (SMS) text messages.

**Objective:**

This study aims to explore insights about the feasibility and practicalities of the utilization of SMS text messaging-based interventions in disease-reporting systems by identifying potential challenges and barriers in the text messaging process and looking at lessons learned.

**Methods:**

An SMS text messaging-based disease tracking system was set up in Vietnam with patient reports texted by clinic staff. Two 6-month trials utilizing this disease tracking system were designed and implemented in two northern provinces of Vietnam to report two infectious diseases: diarrhea and influenza-like illness. A structured self-reported questionnaire was developed to measure the feasibility and practicalities of the system from the participants. On the completion of the second trial in 2013, participating health staff from 40 commune health centers in the two pilot provinces were asked to complete the survey (N=80).

**Results:**

Most participants were female (61%, 49/80) and nearly half (44%, 35/80) were heads of a commune health center. Approximately two-thirds (63%, 50/80) of participants retained the basic structure of the SMS text message report and there was a strong influence (OR 28.2, 95% CI 5.3-151.2) of those people on the time they spent texting the information. The majority (88%, 70/80) felt the information conveyed in the SMS text message report was not difficult to understand. Most (86%, 69/80) believed that they could report all 28 infectious diseases asked for by the Ministry of Health by using SMS text messaging.

**Conclusions:**

From a health center staff perspective, a disease-reporting system utilizing text messaging technology is easy to use and has great potential to be implemented and expanded nationwide. The survey showed positive perceptions and feedback from the participants and contributed to a promising practical solution to improve the surveillance system of infectious disease in Vietnam.

## Introduction

### Potential of Using Text Messaging in Health Care Systems

The use of mobile and wireless technologies to support the delivery of health care services has the potential to transform the face of global health service delivery [1]. In recent years, the number of developing countries that are using mobile technology to address health needs is growing [2]. This is seen especially in African countries; since 2014, a phone-based system for timely integrated disease surveillance and response in Rwanda has collected information on 23 infectious diseases from more than 50% of health facilities nationwide [3]. In 2007, Madagascar launched the first nationwide sentinel surveillance system for influenza-like illness (ILI) based on the use of mobile phones [4]. In Uganda, the feasibility and benefits of a short message service (SMS) text messaging-based reporting system have been demonstrated to help program managers monitor malaria in real time [5].

Mobile phones are becoming cheaper and increasingly accessible. By the end of 2013, there were 97 mobile phone subscriptions per 100 people worldwide. In Vietnam, the number of subscriptions was much higher than many other countries with 131 subscriptions per 100 people [6]. Given the wide coverage of mobile phone services and the low cost of SMS text messaging use in Vietnam, and the fact that the majority of community health centers in Vietnam do not yet use computers to provide their services, there is a great potential for the affordable provision of health services via mobile phones.

### The Infectious Disease Surveillance System in Vietnam

The Vietnamese health care system is hierarchically organized into four administrative levels: central, province, district, and commune. At the central level, the Ministry of Health (MOH), assisted by the National Institute of Hygiene and Epidemiology (NIHE) and other regional Pasteur Institutes [7], is the main national authority in the health sector and is responsible to formulate and implement national health policies and programs. At the provincial level, within each province there are Provincial Health Departments and Preventive Health Centers administered by the Provincial People’s Committee. At the district level, the District People’s Committee administers district health centers and district-level hospitals. A commune health center is a health facility at the lowest level, within each commune, and serves as a primary access point for public health and preventive care services [8,9]. Each commune health center typically provides services for an average of 5000 to 10,000 inhabitants in the commune and has only approximately five to six health care staff members, including one physician—usually the head of the commune health center—and four to five other health professionals, such as assistant physicians, nurses, midwives, and/or pharmacists. Due to the limited human resources for health service provision, one commune health center staff member would have to hold multiple duties and responsibilities; for example, a pharmacist or a nurse might be responsible for checking patients in and out in a paper logbook and reporting disease (a duty that a nurse is normally responsible for).

**Figure 1:**
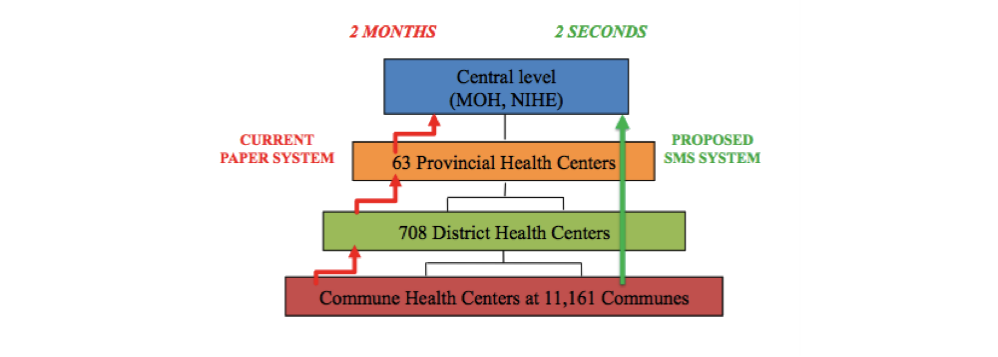
The flow of infectious disease information through the current paper-based reporting system (left-hand side) and the proposed SMS text message-based reporting system (right-hand side).

Infectious disease data in Vietnam are collected through a paper-based system in which data transfers through the four previously mentioned government tiers. Initiated at the commune health center, disease data (eg, cumulative incidence or mortality rates) are aggregated regularly from the patient logbook. The frequency of this commune-level data aggregation depends on the types of diseases, for example, monthly for ILI and diarrhea [10]. Information is then sent to higher administrative levels, to district health centers, then on to preventive health centers, ultimately arriving at NIHE for aggregation and analysis before being reported to the MOH [10]. As a result, through this reporting system, it usually takes 2 months or longer for a final report to reach the MOH (see the left-hand side of Figure 1).

This delay in disease reporting often defers the timely collection of the most recent disease data and contributes to longer government response times. Therefore, it has the potential to be catastrophic in emergency situations such as outbreaks. On the other hand, in addition to a heavy regular workload, the commune health center staff are required to report on a total of 28 infectious diseases [10]. In the effort to improve this, the MOH tested an Internet-based infectious disease monitoring and reporting system in seven provinces in 2012 [11] and officially launched this nationwide in 2014 [12]. However, the effectiveness of the current system remains limited for several reasons, including the fact that many commune health centers are not equipped with computers, have limited access to the Internet, and the system does not provide live trends of disease data.

In 2012, the Institute of Population, Health and Development, in collaboration with the NIHE, Dartmouth College, University of California, Los Angeles, and Columbia University, designed and piloted the use of text messaging for infectious disease surveillance in the public health care system in Vietnam [13,14]. Two 6-month trials were carried out in two northern provinces of Vietnam. A self-reported questionnaire survey was conducted at the end of the second trial in 2013 to evaluate and assess the feasibility and practicalities of the utilization of SMS text message-based disease-reporting system from the commune health center staff’s perspective.

## Methods

### Study Settings

Two northern provinces of Vietnam—Hung Yen and Hoa Binh—were chosen to participate in the study. The first trial was carried out in a total of 20 communes from four districts: Hung Yen city and Yen My district in Hung Yen province, and Hoa Binh city and Ky Son district in Hoa Binh province, 5 communes each. The second trial was carried out in a total of 40 communes located as follows: 17 communes in Yen My district, Hung Yen province (a delta region); 13 communes in Cao Phong district, Hoa Binh province (a mountainous region); and 10 communes in Ky Son district, Hoa Binh province (a mountainous region).

In both trials, the commune health centers’ heads selected and appointed two commune health center staff members from each of the participating communes to participate in the study.

### Study Design

A central data repository server, monitored and managed by NIHE and the research team, was set up. The server was equipped with an SMS text message gateway to collect text messages through a designated cell phone number. In this study, the commune health center staff members were asked to text selective disease information for each record from the patient logbook in a structured but simple format to this cell phone number and make notes of successfully reported cases. Patients diagnosed to have either of the two commonly seen diseases in northern of Vietnam (diarrhea and ILI) were chosen to be reported. The disease data were then electronically collected and stored on the data server where data were summarized and analyzed further to generate the necessary reports. For the purposes of monitoring, the data were also made available on a data management website built on the server, which was securely accessible through the Internet. Only authorized personnel were able to access this data. The right-hand side of Figure 1 illustrates the flow of disease information performed by the proposed SMS text message-based reporting system: disease data were collected at the central level after just a few seconds and were available immediately for different government tiers through the previously mentioned website.

The two trials were implemented for a period of 6 months from July to December in both 2012 and 2013. Accuracy of the proposed text messaging reporting system was examined by aggregating disease data from SMS text message reports, summarizing, and comparing to data from the paper-based reports in the same period. An assessment survey was developed and all commune health center staff participating in the second trial in 2013 completed the survey. All related costs associated with the text messaging process in the study, including the cost of sending SMS text message reports, were covered by the project.

### The Structure of the Text Message Report

**Figure 2:**
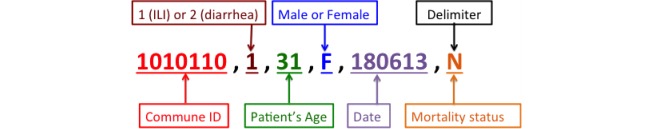
The structure of the SMS text message report in the first trial.

The SMS text message was structured to contain a certain number of simple encoded disease information inputs separated by a designated delimiter. In the first trial, six chosen inputs were commune identification number (assigned uniquely by the research team), disease diagnosis (1 for ILI or 2 for diarrhea), patient’s age, patient’s gender, date of diagnosis, and mortality status. The delimiter was the comma character (see Figure 2). In the second trial, the same text messaging structure was used in the reported SMS text message, but with three different compositions tested in three mentioned districts. One district was asked to send six inputs: the first five were the same as in the previous trial plus ethnic group in lieu of mortality status for the last input. One district was asked to send in only three inputs: commune identification number, disease diagnosis, and date of diagnosis. The remaining district was asked to send in 11 inputs: commune identification number, disease diagnosis, patient’s age, patient’s gender, date of diagnosis, village, patient’s occupation, patient’s ethnic group, disease symptoms, treatment provided, and name of physician. In the second trial, a space character was used as a delimiter.

### Training and Monitoring

Face-to-face training classes, focusing on SMS text message sending techniques and some typical mistakes, such as mistyping or duplicate reports, were provided to all the participants in preparation for the implementation of each of the trials. Two participating commune health center staff members from each commune health center, who were appointed and registered with the study by the commune health center’s head, attended the training. Handouts and leaflets about the study were also provided to the commune health center staff members at the training.

To minimize mistyping, typos, or duplicate SMS text message reports, the data were monitored closely through the data management website by a project officer. Daily SMS text message reports were randomly checked and any mistyping or doubtful report was reported by phone to the corresponding commune health center staff member for double-checking, fixing, and resending if necessary. On-site monitoring trips were also organized every 3 months during the implementation of the project to some randomly chosen commune health centers, where the research team checked with the commune health center staff members to make sure the protocol of the project was followed precisely, for example, commune health center staff compliance with the SMS text message report sending instructions or consistency between the patient logbook and the SMS text message reports on any random date.

### Survey Development and Data Collection

A structured self-reported questionnaire was developed to measure the feasibility and practicalities of the SMS text message-based reporting system. Information was collected including (1) general and demographic information of participants, such as gender, ethnicity, or position at the commune health center; (2) characteristics of SMS text message-based surveillance system, such as structure of SMS text message reports, time spent sending SMS text messages, or their thoughts about the system; (3) technical issues that arose during the texting process, what issues they met when sending messages, and how they dealt with the situation; (4) facilities at the commune health center, such as computer and Internet capabilities; and (5) participants’ comments and suggestions.

The survey was conducted in January 2014 after the second trial ended. All 80 participating commune health center staff members from 40 communes were asked to anonymously complete the survey.

### Statistical Analysis

Data entry was conducted using EpiData software [15] and data analysis was performed using Stata version 12.0 (StataCorp LP, College Station, TX, USA). Quantitative descriptive analysis, Pearson chi-square test, and multivariate logistic regression were used to examine different relationships and correlations from the survey data. A *P* value of <.05 was considered statistically significant.

### Ethical Consideration

The activities in this study, which were reviewed and received MOH approval before the implementation, were considered to be part of the daily duty of the participating commune health center staff members. The study was designed to collect only patients’ general information for statistical purposes. Access to the repository server, as well as the SMS text message reports and databases, were strictly controlled and only permitted authorized personnel. The questionnaire survey was developed to not contain any identifiable information from the participants and was conducted anonymously.

## Results

### Demographic and General Information

Most participants were female (61%, 49/80). Nearly half (44%, 35/80) were heads of commune health centers. One-third (34%, 27/80) of participants were ethnic minorities from mountainous areas in Cao Phong and Ky Son districts (see Table 1).

Overall, 29 of 80 participants (36%) took part in the first trial. Of these, 17 participants were from Ky Son district followed by 12 participants from Yen My district. Cao Phong district was new to the second trial. Only one participant (1%, 1/80) could not take part in the training for the second trial. Here, their colleagues successfully guided them on how to send the SMS text message report. The majority of participants (93%, 74/80) reported that they shared information on the project with other staff members. A mean 2.3 (SD 1.0) staff members in each of the 40 communes participated in the second trial (by district Cao Phong: mean 2.8, SD 0.8; Ky Son: mean 2.2, SD 1.7; Yen My: mean 2.0, SD 1.0).

When sending SMS text message reports, 73 of 80 participants (91%) used the Viettel network, whereas a small number of participants used other networks, such as Vinaphone (8%, 6/80) and Mobiphone (1%, 1/80).

### Practicalities of the System

Overall, 50 of 80 participants (63%) retained the basis of the required structure and components of an SMS text message report. This basic knowledge included three formatting requirements of an SMS text message report: the number of data inputs to be reported (3, 6, or 11), incidents per report (1), and the symbol used to separate the data inputs in the report (the delimiter, either a comma or a space). Cao Phong, a new district for the second trial, had the highest percentage of participants retaining the basis (88%, 23/26) among the three districts.

A detailed breakdown of the percentage of people retaining the basic structure of an SMS text message report into three formatting components is shown in Table 2. Regarding the number of data inputs to be reported, all participants in Cao Phong district (100%, 26/26), who were asked to send in six data inputs, recalled this number compared to Yen My (three inputs) with 94% (32/34). Participants in Ky Son district, who sent in the highest number of data inputs (11), had the lowest recall (75%, 15/20). When other components of the SMS text message format were examined (eg, incidents per message and the delimiter), Cao Phong still came out on top.

**Table 1:** Demographic information of participants.

Demographic information		Hung Yen (delta region), n (%)	Hoa Binh (mountainous region), n (%)		Overall, n (%) (N=80)
		Yen My (n=34)	Cao Phong (n=26)	Ky Son (n=20)	
**Gender**					
	Male	12 (35)	12 (46)	7 (35)	31 (39)
	Female	22 (65)	14 (54)	13 (65)	49 (61)
**Ethnicity**					
	Kinh	34 (100)	12 (46)	7 (35)	53 (66)
	Ethnic minority	0	14 (54)	13 (65)	27 (34)
**Position in commune health center**					
	Head/physician	14 (41)	11 (42)	10 (50)	35 (44)
	Nurse/assistant physician	14 (41)	3 (12)	3 (15)	20 (25)
	Pharmacist	4 (12)	1 (4)	1 (5)	6 (7)
	Other	2 (6)	11 (42)	6 (30)	19 (24)

**Table 2:** Percentage of participants retaining the three formatting structures of an SMS text message report: number of data inputs, incidents per report, and delimiter.

Basic structure of an SMS text message report	Yen My, n (%) (n=34)	Ky Son, n (%) (n=20)	Cao Phong, n (%) (n=26)	Overall, n (%) (N=80)
Number of data inputs	32 (94)	15 (75)	26 (100)	73 (91)
Incidents per SMS text message	22 (65)	13 (65)	23 (88)	58 (73)
Delimiter	22 (65)	19 (95)	26 (100)	67 (84)
Basic knowledge (all three formatting components above)	17 (50)	10 (50)	23 (88)	50 (63)

Overall, 88% (70/80) of participants felt that the information required to be reported was not difficult to text and 95% (76/80) of participants felt that no information should be removed from the messages, 89% (71/80) thought that no further information needed to be added, but a small number (5%, 4/80) suggested disease severity should be included. Regarding the adequacy of disease information to be reported per message, only a small number of participants thought the amount of information in the SMS text message was too much (6%, 5/80) or not enough (9%, 7/80).

Of the 80 participants, 55 (69%) agreed with the use of a space to separate each piece of information in an SMS text message, whereas some preferred using a period (18%, 14/80) or a comma (10%, 8/80) or no response (4%, 3/80). One-third (26/80) said that they usually sent disease cases after having the diagnosis confirmed, whereas 64% (51/80) reported they sent the SMS text message at the end of day.

The majority of participants (84%, 67/80) reported they did not spend much time sending messages, whereas 13 participants (16%, 13/80) felt that they needed more time. Of these, most (62%, 8/13) were from Yen My district, who needed to send in three data inputs, and from Ky Son (39%, 5/13), a district with 11 data inputs.

**Figure 3:**
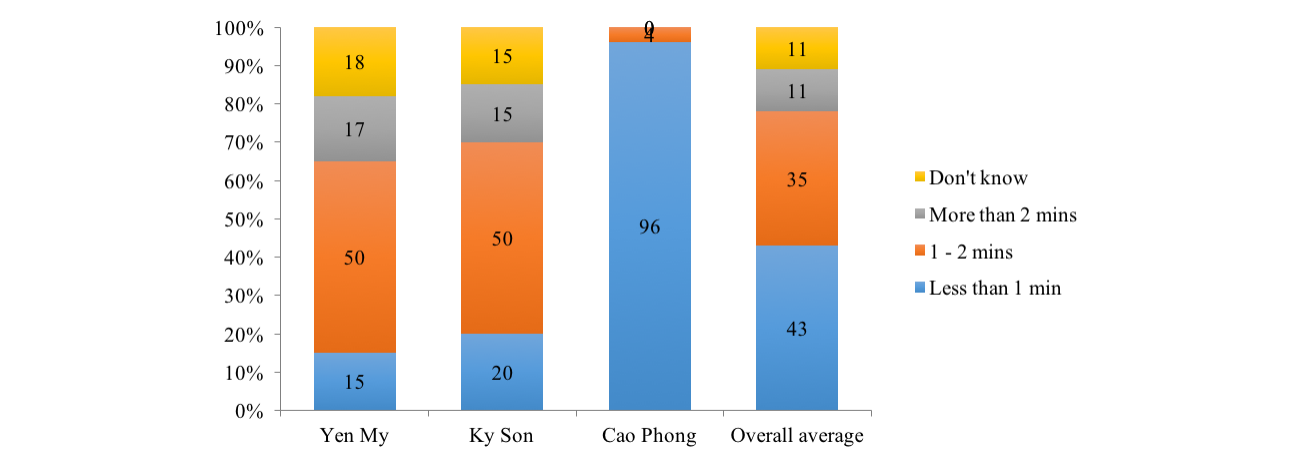
Time spent sending an SMS text message report by district.

Regarding the time spent for performing the work, less than half of the participants (43%, 34/80) said they needed less than 1 minute to text a message, whereas some (11%, 9/80) said they needed more than 2 minutes (see Figure 3). The time spent sending the SMS text message was not significantly different between male and female participants (*P*=.15).

A multivariate logistic regression performed to study the influence of ethnicity, sex, and retaining the basic structure of an SMS text message report on the participants’ report of taking less time to send SMS text message reports showed that there was a significant difference between different ethnic groups (OR 14.7, 95% CI 1.5-139.4) in which ethnic minority participants needed less time to text than Kinh participants (see Table 3). In Cao Phong district, all ethnic minority participants (100%, 14/14) needed less than 1 minute to send an SMS text message report compared with 92% (11/12) of Kinh participants, who needed 2 minutes or more. In Ky Son, three of five Kinh participants (60%) said they needed more than 2 minutes to send an SMS text message compared to three of 13 ethnic minority participants (23%). The rates for less than 1 minute were similar (Kinh: 20%, 1/5; ethnic minority: 23%, 3/13).

We also found a strong influence (OR 28.2, 95% CI 5.3-151.2) on the time spent sending an SMS text message for the participants who retained the SMS text message report’s basic knowledge. A multivariate logistic regression model to study this influence on each of the components of the SMS text message report’s basic structure found that although all three components had significant influence, the delimiter had the strongest impact on people who spent less than 2 minutes to send an SMS text message report (see Table 4).

**Table 3:** Multivariate logistic regression model on the participants’ report of taking less time to send SMS text message reports.

Variables		OR (95% CI)	*P*
**Basic structure of an SMS text message report**			
	Do not remember	1	
	Remember	28.2 (5.3-151.2)	<.001
**Sex**			
	Male	1	
	Female	0.7 (0.1-3.6)	.67
**Ethnicity**			
	Kinh	1	
	Ethnic minority	14.7 (1.5-139.4)	.02

**Table 4:** Multivariate logistic regression model to study the influence of each component of the basic SMS text message report’s structure on time spent to send an SMS text message report (less than 2 minutes to send).

Variables		OR (95% CI)	*P*
**The number of data inputs**			
	Do not remember	1	
	Remember	10.7 (1.4-79.9)	.02
**Incidents per SMS text message**			
	Do not remember	1	
	Remember	4.9 (1.3-19.4)	.02
**Delimiter**			
	Do not remember	1	
	Remember	13.2 (2.8-61.6)	.001

Information for all patients who visited the commune health center was recorded in a logbook. When sending an SMS text message report, commune health center staff were asked to make notes in the logbook to avoid duplicate reporting. A vast majority of participants (91%, 73/80) reported that they recorded reported cases in a logbook. Most participants (86%, 69/80) believed that they could report all 28 infectious diseases by using the SMS text message system. It was reported by the commune health center staff members that, on average, they saw a mean 6.9 (SD 3.2) patients per day (by district Cao Phong: mean 8.1, SD 2.7; Ky Son: mean 5.5, SD 3.3; Yen My: mean 6.9, SD 3.2).

When participants were asked about what ongoing support they would like to assist them with their work, half said they would like face-to-face refresher training (45%, 36/80) followed by handouts (39%, 31/80) and videos (19%, 15/80). When we looked at the two-group comparison, as shown in Table 5, ethnic minority participants (60%, 15/25) preferred refresher training compared to Kinh participants (43%, 19/44), who had a preference for posters and videos. This reflects the practicality of this type of work and the usefulness of having someone on the spot who can give hands-on guidance, answer questions, and solve problems quickly.

**Table 5:** Types of support requested by the participants.

Types of support	Kinh, n (%)	Ethnic minority, n (%)	Total, n (%)
Poster	10 (20)	1 (4)	11 (14)
Video	11 (22)	3 (11)	15 (19)
Handout	22 (44)	9 (33)	31 (39)
Refresher training	19 (38)	15 (56)	36 (45)
Other	10 (20)	2 (7.4)	12 (15)

### Technical Issues

Half of the participants (40/80) reported that sometimes they were not able to send the SMS text message to the server. Of these, 45% (18/40) said that this occurred rarely or sometimes (55%, 22/40). “No signal” was the biggest reason seen for this problem (65%, 26/40) followed by server issues (25%, 10/40) and unknown (10%, 4/40). Most of the “no signal” issues (65%, 17/26) were reported by health staff in Cao Phong district—a mountainous area. Where there were difficulties sending the SMS text message the first time, the majority (78%, 31/40) decided to send it again a later time. Less than half (40%, 16/40) decided to use another mobile phone to do this. Only a few (15%, 6/40) called the project team to ask about this issue.

### Feasibility of the System

To assess the feasibility of the use of text messaging in a disease surveillance system, we performed multivariate logistic regression analysis to study the impact of the SMS text message report’s basic knowledge, the adequacy of disease information per SMS text message report, and the technical issues on the possibility of reporting all 28 infectious diseases by text messaging. The result showed that the SMS report’s basic knowledge alone highly influenced the possibility of reporting all 28 infectious diseases by text messaging (see Table 6). A deeper look at the effect of each of the components of the SMS text message report’s structure showed that although the incidents per SMS text message report and the delimiter had high influence (see Table 7), the number of data inputs per SMS text message report had no significant association (*P*=.30).

**Table 6:** Multivariate logistic regression model on the possibility of reporting all 28 diseases asked for by the MOH.

Variables		OR (95% CI)	*P*
**Basic structure of an SMS text message report**			
	Do not remember	1	
	Remember	8.6 (1.6-45.6)	.01
**Disease information per SMS text message report**			
	Insufficient	1	
	Sufficient	0.9 (0.1-9.4)	.91
**Technical issues when sending an SMS text message**			
	No	1	
	Yes	0.8 (0.2-3.7)	.82

**Table 7:** Multivariate logistic regression model to study the impact on the possibility of reporting all 28 diseases asked for by the MOH of different components of the SMS text message report.

Variables		OR (95% CI)	*P*
**Incidents per SMS text message**			
	Do not remember	1	
	Remember	6.4 (1.4-29.6)	.02
**Delimiter**			
	Do not remember	1	
	Remember	5.2 (1.1-24.6)	.04

### Commune Health Center Internet Capabilities

The survey also investigated the computer and Internet capacity of the participating commune health centers. More than half (56%, 45/80) of commune health center staff reported that their health center was equipped with a computer; however, only 49% (22/45) had an Internet connection and, among those, most (82%, 18/22) said that their center had the budget to maintain it. Of the commune health centers that had a computer but no Internet access, 96% (22/23) were located in areas with the availability of broadband Internet services. Less than half (41%, 33/80) had the budget to cover the cost of this. For those commune health centers without a computer, Internet services were available for most of them (95%, 32/34), but only 26% (8/31) could afford the cost.

### Input From Commune Health Center Staff

All participants were asked to provide their comments or suggestions to improve the SMS text message-based surveillance system. We received comments from 23 of 80 participants (29%). Of these, nine participants (11%) said that the system should be maintained and expanded to a larger scale, for example, provincewide or nationwide. Six participants (8%) wanted to have a feedback mechanism in the system to help them perform their work better. This included checking to see if there were any mistakes in the report or if their report had been sent successfully. In addition, five participants (6%) wanted to be provided computer and network facilities.

## Discussion

### Principal Results

The study showed that basic structure of an SMS text message report plays an important role in both the practicalities and feasibility of a pilot texting system. This includes a strong influence on the time spent to text a report and a high impact on the ability to send in all 28 infectious diseases as required by MOH using SMS text message reports. This also means education and training prior to commencement of the system, in combination with refresher training and other information, education, and communication materials, such as leaflets or posters, during the implementation are critical to the success of the project.

In terms of demographics, despite only two staff members per commune health center participating in the study, the sex ratio of the participants reflects the domination of female health staff working in health care facilities in Vietnam [16]. A mix of different health center staff positions, especially the commune health center’s head, participating in the study may indicate the human resource limitation in health care in Vietnam, in which a staff usually has to hold multiple duties and responsibilities in their center.

One-third of participants in the study were ethnic minority who needed less time to text than Kinh participants did. This may demonstrate that participant’s interest in a new and novel project could be a factor that influences the performance of their work. Ethnic minority participants, who live in remote and mountainous areas where health care services are limited, may be more interested and motivated about the use of health care technologies that might bring more benefit for them than Kinh participants. The outperformance of participants from Cao Phong district—a new district that had not been previously exposed to the project—may have also contributed to this point.

In terms of texting skills or the time spent to send an SMS text message, a strong relation between this and the SMS text message’s structure basis showed that people who were fully engaged in the project were quicker and more effective in composing and texting messages. Therefore, skills in using a mobile phone along with concentration can be factors related to the time needed to send an SMS text message. That there was no significant difference in the time spent sending SMS text messages between male and female participants is intriguing. This finding does not reflect some recent studies on time spent by males and females in sending an SMS text message, where it was reported that women tended to spend less time than men [17,18], potentially due to women having smaller fingers enabling them to type faster and more easily [17].

In terms of technical issues, “no signal” came out on top among the reasons causing the inability to send an SMS text message. This is an ongoing challenge, especially for remote and mountainous areas in Vietnam, and ways of overcoming and mitigating these kinds of technical events to maintain commitment to the SMS text message reporting process include discussion with the mobile phone providers. However, our study showed that these texting technical issues do not affect the feasibility of the reporting system from the participant’s perspective. Additionally, the study offered some creative ways invented by the participants to temporarily solve the issues, such as sending the message again or using another phone.

In terms of data accessibility and availability, the computer and Internet capacity of the commune health center would be useful for the participants to learn more about the project’s information, to refresh their knowledge, and especially to create reports from the live data. However, computers and Internet are not fully equipped in all communes. This presents a challenge in terms of staff accessing data and transmitting reports electronically. The necessary funding support will be required to make all commune health centers fully operational.

### Limitations

The survey did not cover inputs from the district, provincial, and national levels, which might provide more valuable information, such as on the administrative aspect of the project. Disease data aggregated from the pilot system and its comparison to a paper-based system and the statistics and problems of SMS text message reports, such as mistyping or duplicates, which would certainly contribute to a much stronger assessment of the system, was not discussed. In addition, the study was limited by its small sample size and includes a nonrandomized, uncontrolled single-arm study design that possibly precludes causal inference of the SMS text message-based report system to the outcomes.

### Conclusions

Despite being a new disease-reporting system involving modern technology and knowledge, the SMS text message-based disease surveillance system demonstrated its ease of use and received positive feedback from participants. It is especially important that this work does not take too much time away from the health center staff’s other duties. Although more in-depth studies might be needed to assess whether the information collected is sufficient for live reporting and outbreak prediction, the project showed its great potential to be implemented and expanded on a larger scale, such as provincewide or nationwide.

In addition to its perception and feasibility, the project provided valuable learning lessons, including regular refresher training for the commune health center staff, which is critical not only to enhance participants’ engagement in the project, but also to improve the precision of reported data and to help solve technical problems. Well-prepared training, using a combination of lessons learned and experience gained from the previous trial, will also help a great deal in designing a more effective bidirectional instruction and training for the participants.

Some technical lessons were also learned from the two trials. First, improved software, which can recognize multiple delimiters, would eliminate the difficulties arising from the commune health center staff member’s texting habits of using a comma (,) or period (.) to separate the reported piece of information. Second, an integrated feedback mechanism not only to address errors and concerns, but also to give disease information back to the health care worker will help in screening any mistake or typo in the reported SMS text message. Finally, a live disease trend map (or disease heat map) and a timely reporting system will help not only the high-level administration, such as MOH and NIHE, to make appropriate decisions and take the required actions in case of outbreak, but also the provincial, district, and commune staff in monitoring their work and generating reports when required.

## Abbreviations

ILI: influenza-like illness
MOH: Ministry of Health
NIHE: National Institute of Hygiene and Epidemiology
SMS: short message service
